# Supplementary material for: Cytoophidia Maintain the Integrity of Drosophila Follicle Epithelium
Source: Int J Mol Sci. 2022 Dec 4;23(23):15282. doi: 10.3390/ijms232315282 (PMC9740582; doi:10.3390/ijms232315282)
Supplement: Supplementary file 1 [file ijms-23-15282-s001.zip › ijms-2001317-supplementary.pdf]

# Supplementary Figure S1 and Figure Legends

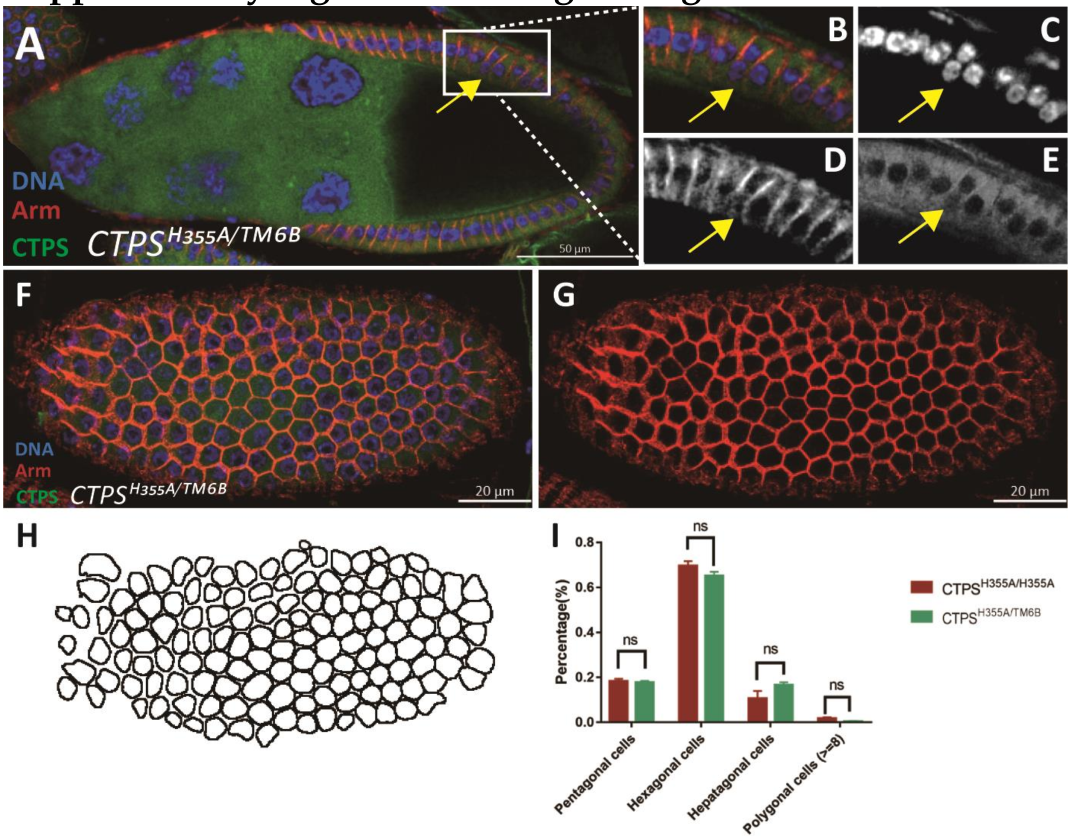


**Supplementary Figure S1. *CTPS$^{H355A/TM6B}$* mutants have no siginificant difference from *CTPS$^{H355A}$* mutant.** (A-E) Follicle cell ingression in *CTPS$^{H355A/TM6B}$* mutants. CTPS is labelled using CTPS antibody and there is no cytoophidia (Green), Armadillo marked cell membrane (Red), DNA is labelled using HOECHST 33342 (Blue). (F-H) Surface view of a stage 8 *CTPS$^{H355A/TM6B}$* egg chamber. (I) Quantitative analysis of the morphological difference between the *CTPS$^{H355A}$* and the *CTPS$^{H355A/TM6B}$* follicle cells (6 egg chambers were quantified at stage 8 per genotype, biological repeats = 3). Mann Whitney U test.
